# Expression of nucleotide-binding oligomerization domain-like receptor protein 1 in patients with acute myocardial infarction and its relationship with clinical prognosis

**DOI:** 10.3389/fcvm.2025.1685953

**Published:** 2025-10-20

**Authors:** Wenhao Qian, Chang Liu, Zheng Chen, Di Wu, Jianhua Wang, Han Yao, Fangfang Li

**Affiliations:** ^1^ Department of Cardiology, The Affiliated Hospital of Xuzhou Medical University, Xuzhou, China; ^2^ Department of General Practice, The Affiliated Hospital of Xuzhou Medical University, Xuzhou, China; ^3^ Xuzhou Medical University, Xuzhou, China

**Keywords:** NLRP1, acute myocardial infarction, inflammation, MACE, biomarker

## Abstract

**Background:**

Acute myocardial infarction (AMI) remains the leading cause of cardiovascular-related mortality worldwide, with inflammation significantly influencing its progression and prognosis. Nucleotide-binding oligomerization domain-like receptor protein 1 (NLRP1), a key inflammasome regulator, facilitates the release of pro-inflammatory factors. However, its expression profile in AMI and its relationship with inflammation and prognosis are not well understood.

**Methods:**

A total of 245 AMI patients (undergoing emergency percutaneous coronary intervention within 12 h), 60 patients with unstable angina (UA), and 60 healthy controls were included. Serum NLRP1 levels were detected by enzyme-linked immunosorbent assay, and clinical indicators were measured. The AMI patients were followed up for 6 months to record major adverse cardiovascular events (MACE). Correlation analysis, regression models, and receiver operating characteristic (ROC) curves were used to evaluate its prognostic value.

**Results:**

Serum NLRP1 levels increased with the severity of the disease (healthy controls < UA < AMI, *P* < 0.05) and were significantly correlated with inflammatory markers [such as high-sensitivity C-reactive protein and systemic inflammatory response index (SIRI)] and myocardial injury markers [such as high-sensitivity cardiac troponin T (hs-cTnT)] in AMI patients (*P* < 0.05). A 6-month follow-up showed that AMI patients with MACE had higher NLRP1 levels (*P* < 0.001), and NLRP1 was an independent risk factor for MACE (odds ratio = 1.01, *P* = 0.013). Stratified analyses showed that NLRP1 added predictive value, particularly in patients with low hs-cTnT and low SIRI, improving the area under the curve (AUC) (*P* < 0.05). The ROC curve indicated that NLRP1 alone had an AUC of 0.718 for predicting MACE, which increased to 0.822 when combined with traditional markers (*P* < 0.05). Category-free net reclassification improvement (NRI) analysis showed a significant improvement in risk reclassification (NRI = 0.315, *P* = 0.037).

**Discussion:**

Serum NLRP1 levels correlate with coronary heart disease severity, indicating inflammation and myocardial injury, and independently predict short-term MACE in AMI. When combined with traditional markers, NLRP1 enhances prognostic assessment efficiency and holds potential as a novel inflammatory marker.

## Introduction

Acute myocardial infarction (AMI) is the leading cause of the incidence and mortality of cardiovascular diseases worldwide (1, 2). Most cases of AMI occur based on coronary artery disease (CAD), where a sudden reduction or interruption of the coronary blood supply leads to severe and persistent myocardial ischemia and hypoxia, ultimately resulting in myocardial necrosis (3). Despite advancements in treatments such as drug therapy and revascularization that have lowered adverse outcomes of AMI, the prognosis for AMI patients remains poor, with a substantial risk of MACE (4). Early diagnosis, treatment, risk analysis, and prognosis assessment are therefore clinically important. Inflammation is pivotal in the onset and progression of AMI, contributing to myocardial injury, plaque instability, and adverse ventricular remodeling. The intensity and duration of the inflammatory response are closely linked to the prognosis of AMI patients (5, 6).

Nucleotide-binding oligomerization domain-like receptor protein 1 (NLRP1), a pivotal regulatory molecule in the inflammasome complex, facilitates the maturation and release of pro-inflammatory factors like IL-1β and IL-18 through caspase-1 activation, thus playing an essential role in inflammation-related diseases (7, 8). In clinical studies, the expression level of NLRP1 has been found to be closely associated with the clinical phenotypes of multiple inflammation-related diseases. In patients with unstable angina (UA), the NLRP1 level shows a significant positive correlation with the severity of coronary artery lesions (Gensini score), and patients with high NLRP1 expression have a higher incidence of major adverse cardiovascular events (MACE) after surgery than those with normal expression. However, the expression characteristics of NLRP1 in patients with AMI, its specific associations with the intensity of the inflammatory response and the risk of MACE, and whether it can serve as a novel inflammatory marker for optimizing the prognostic assessment of AMI have not been systematically elucidated, which also becomes the core direction of this study.

To further contextualize our findings on NLRP1 and inflammation in AMI, it is important to note the role of systemic inflammatory assessment in our study design. While single inflammatory markers [e.g., high-sensitivity C-reactive protein (hs-CRP) and white blood cell count] were included in multivariable analyses to capture specific aspects of inflammation, we also incorporated composite indices—the systemic inflammatory index (SII) and systemic inflammatory response index (SIRI)—to achieve a more comprehensive evaluation of systemic inflammatory imbalance. These indices, which integrate immune cell subset interactions (neutrophils, lymphocytes, platelets/monocytes), have been validated as reliable prognostic tools in AMI (9, 10), and their inclusion in relevant statistical analyses supplemented our assessment of inflammatory status.

## Research design

1. Inclusion criteria for AMI patients [undergoing percutaneous coronary intervention (PCI)]:
  • Patients who were admitted to the Affiliated Hospital of Xuzhou Medical University from January 2024 to December 2024 due to AMI and underwent emergency PCI within 12 h
  • Patients aged over 18 years
2. Inclusion criteria for 60 healthy controls during the same period [undergoing coronary angiography (CAG) without a diagnosis of CAD]:
  • Individuals who underwent CAG due to chest pain or other suspected symptoms, with the results showing no coronary artery stenosis or stenosis < 30%, and were not diagnosed with CAD
  • Normal results in cardiac troponin, electrocardiogram, and echocardiogram examinations
  • Age and gender matched with the AMI group (±5 years)
3. Inclusion criteria for 60 patients with UA during the same period (based on CAG results):
  • Diagnosed with unstable angina; CAG confirmed that at least one major coronary artery had a stenosis ≥70% without evidence of acute occlusion
  • Age and gender matched with the AMI group (±5 years)
  • Not receiving emergency PCI treatment (stable condition or elective intervention)
4. Exclusion criteria:
  (1) Severe underlying diseases: comorbid severe liver or kidney failure (creatinine ≥ 3 mg/dL or Child–Pugh class C), malignant tumors, hematological diseases, autoimmune diseases, or chronic inflammatory diseases
  • Recent acute events: a history of severe infection, trauma, or surgery within the past 3 months (AMI group); a history of acute diseases or surgery within the past 6 months (normal control group); progression to AMI or emergency PCI within the past 1 month (UA group)
  • Heart-related diseases: a previously confirmed diagnosis of cardiomyopathy, congenital heart disease, severe valvular disease, pericardial disease, etc.
  • Treatment-related contraindications: allergy to PCI-related drugs (such as aspirin, clopidogrel, and contrast agents), failure to regularly take dual antiplatelet therapy after surgery (specific to the AMI group)
  • Data and follow-up issues: incomplete clinical data, loss of follow-up contact (AMI group); incomplete data (normal population, UA group)

A total of 60 healthy controls (HC), 60 patients with UA, and 245 patients with AMI were finally included. (1) Patients were divided into the healthy control group, UA group, and AMI group according to the diagnostic criteria. (2) AMI patients were followed up for half a year after discharge. Patients were divided into the MACE group (*n* = 59) and the non-major adverse cardiovascular events (NMACE) group (*n* = 186) based on whether MACE occurred within half a year. (3) Based on the cutoff value of the receiver operating characteristic (ROC) curve, AMI patients were divided into the high-NLRP1 group (*n* = 117) and the low-NLRP1 group (*n* = 128).

**Figure 1:**
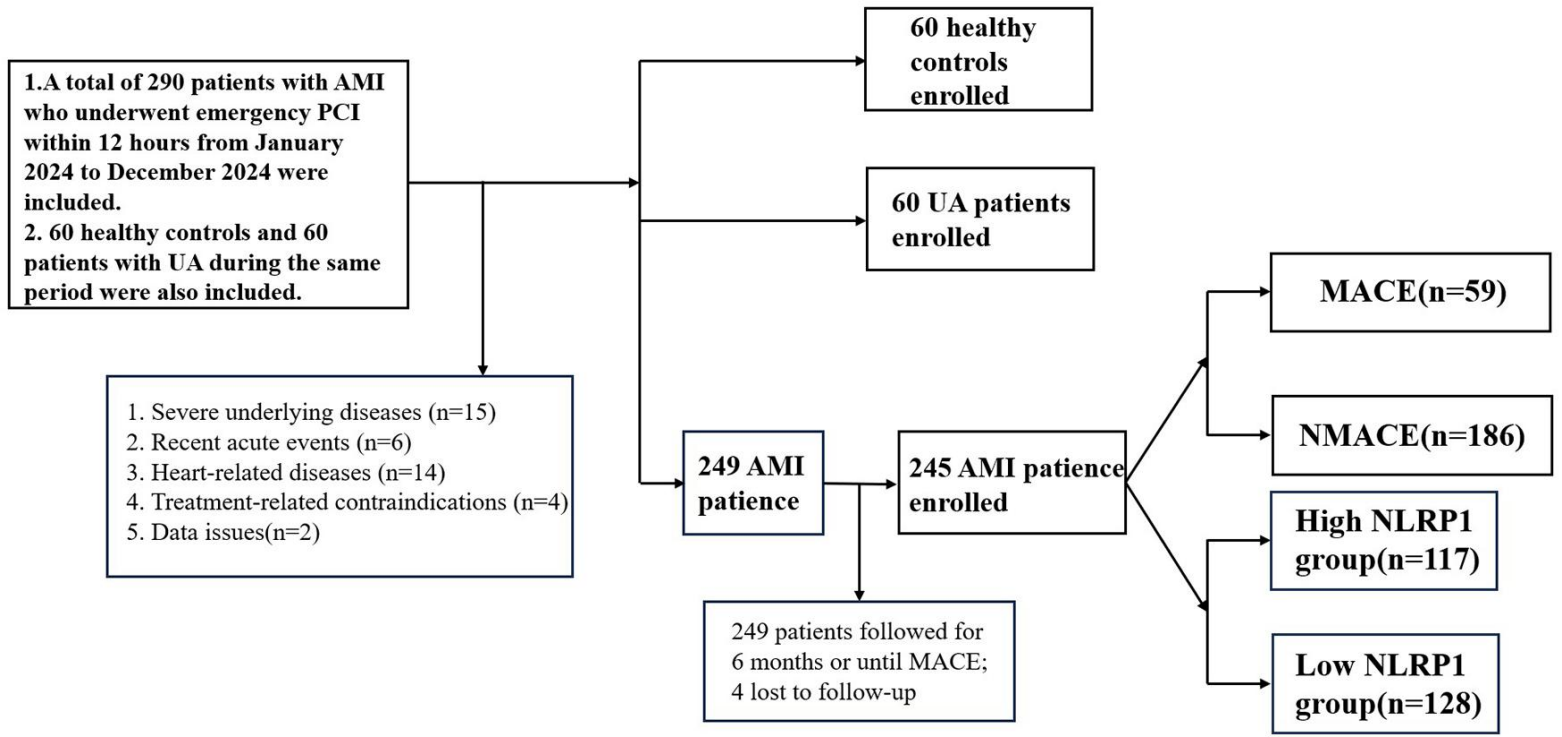
Technical roadmap. AMI, acute myocardial infarction; UA, unstable angina; NMACE, non-major adverse cardiovascular events; MACE, major adverse cardiovascular events; NLRP1, NOD-like receptor protein 1.

Follow-up was conducted once a month after discharge and ended after 6 months. Follow-up methods included outpatient re-examinations and telephone or online software follow-ups, and all occurrences of MACE were recorded (Figure 1).

### Data sources and collection

Venous blood samples were collected from all study subjects within 24 h of admission [except for the peak values of peak hs-cTnT, peak N-terminal pro-brain natriuretic peptide (NT-proBNP), and peak creatine kinase-MB isoenzyme (CK-MB)]. The samples were tested in the biochemical laboratory of the Affiliated Hospital of Xuzhou Medical University, including complete blood count and serum biochemical indicators [total cholesterol (TC), triglyceride (TG), high-density lipoprotein cholesterol (HDL-C), low-density lipoprotein cholesterol (LDL-C), creatinine, etc.]. Electrocardiogram results, blood pressure, height, weight, and past medical histories (such as a history of hypertension, smoking, and diabetes) of the patients at the time of admission were collected. Meanwhile, the enzyme-linked immunosorbent assay (ELISA) (CUSABIO, Wuhan Huamei Bio-Engineering Co., Ltd.) was used to detect the NLRP1 level in the serum of patients with coronary heart disease. The serum NLRP1 level was detected strictly according to the instruction manual.

### Definition

AMI is defined as at least one cardiac troponin value above the 99th percentile upper reference limit with a rising and/or falling pattern together with at least one of the following: (1) symptoms of myocardial ischemia; (2) new ischemic electrocardiographic (ECG) changes (ST–T changes or new pathological Q waves); (3) imaging evidence of new loss of viable myocardium or new regional wall motion abnormality consistent with an ischemic etiology; (4) identification of a coronary thrombus by angiography or autopsy (3).

UA refers to patients presenting with symptoms and/or signs of myocardial ischemia without evidence of acute myocardial injury (i.e., high-sensitivity troponin values remain below the 99th percentile upper reference limit (URL) and without dynamic changes). Transient ischemic ECG changes may be present (11).

MACE: (1) cardiovascular death; (2) non-fatal acute myocardial infarction (recurrence); (3) non-fatal stroke; (4) readmission for heart failure; (5) target vessel revascularization (TVR) (12).

SII = (platelet count × neutrophil count) / lymphocyte count (13).

SIRI = (neutrophil count × monocyte count) / lymphocyte count (14).

### Statistical analysis

Baseline data: Continuous variables were presented as mean ± standard deviation (SD) or median [interquartile range (IQR)], and the *t*-test or Mann–Whitney *U* test was used for intergroup comparisons. Categorical variables were presented as frequency (percentage), and the *χ*^2^ test was used for intergroup comparisons. The Spearman correlation analysis method was used to analyze the correlations between NLRP1 levels and hs-CRP, NT-proBNP, hs-cTnT, CK-MB, SII, SIRI, fibrinogen, and D-dimer in AMI patients. To identify potential predictors of MACE, LASSO regression was first applied for variable selection. Subsequently, bootstrap resampling was used to validate the stability of the selected variables from the LASSO model. Variables with consistent non-zero coefficients across the bootstrap samples were retained for further analysis. These selected variables were then evaluated in a multivariate logistic regression model to identify independent predictors of MACE. Effect sizes for NLRP1 were calculated using odds ratios (ORs) and 95% confidence intervals (CIs). To enhance clinical interpretability, effect sizes were also presented using SD and IQR. The dose–response relationship between NLRP1 and MACE was analyzed using restricted cubic splines (RCS). The sensitivity and specificity of NLRP1 in predicting MACE were evaluated using the ROC curve. The DeLong test was used to compare the area under the curve (AUC) of the combined variables. Stratified analyses were conducted to examine the incremental value of NLRP1 in subgroups defined by systemic inflammatory markers (e.g., SIRI and hs-CRP) and myocardial injury markers (e.g., hs-cTnT and NT-proBNP). To evaluate the clinical utility of adding NLRP1 to established biomarkers, net reclassification improvement (NRI), integrated discrimination improvement (IDI), and decision curve analysis (DCA) were performed. The NLRP1 was divided into high and low groups based on the ROC cutoff value, and the baseline characteristics and the incidence of MACE were compared between the two groups. All statistical methods used a two-sided *P* < 0.05 as the criterion for statistical significance. All statistical analyses and plotting were performed using SPSS 27.0, R 4.2, and GraphPad Prism 9.5.0 software.

## Results

### Comparison of clinical data among normal individuals, UA patients, and AMI patients

**Figure 2:**
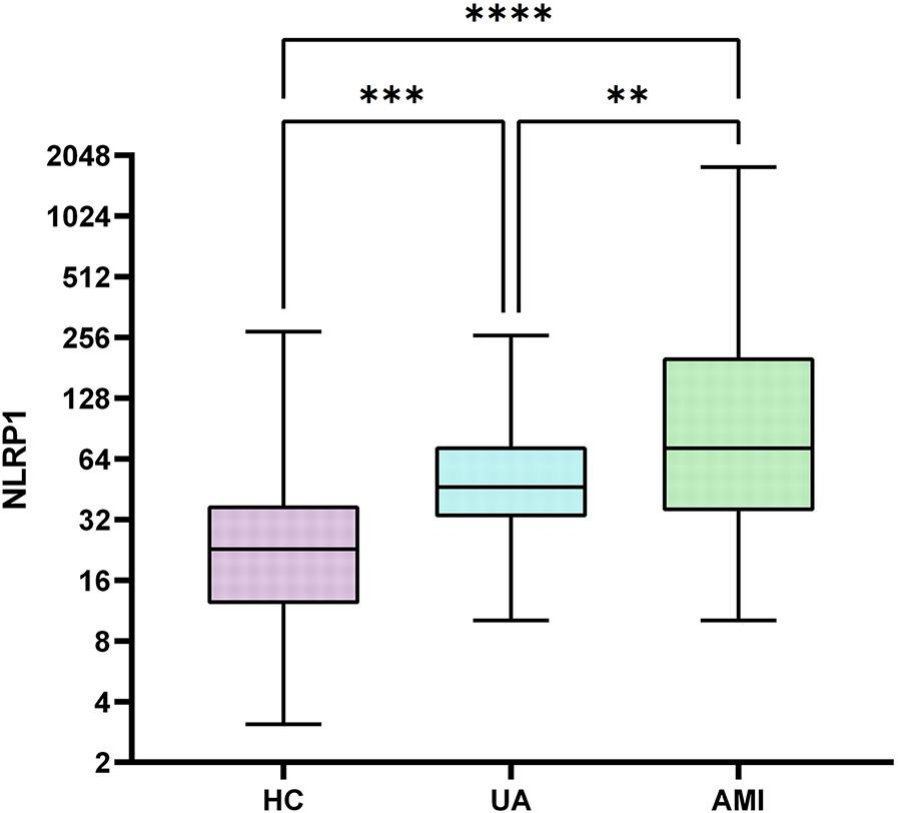
Box plots comparison of NLRP1 levels in three groups. There were significant differences in NLRP1 levels when comparing each two groups among HC, UA, and AMI groups (HC vs. UA, *P* < 0.001; UA vs. AMI, *P* < 0.05; HC vs. AMI, *P* < 0.0001); HC group, healthy control group; UA group, unstable angina group; AMI group, acute myocardial infarction group; NLRPL, NOD-like receptor protein 1.

This study included a total of 345 participants, including 60 healthy controls (HC group), 60 patients with UA (UA group), and 245 patients with AMI (AMI group). There were no significant differences among the three groups in gender distribution (Table 1, with male proportions of 61.67%, 71.67%, and 75.51% respectively, *P* = 0.097) and the prevalence of diabetes (Table 1, 18.33%, 35.00%, and 30.2% respectively, *P* = 0.104). Compared with the other two groups, the AMI group had significantly elevated levels of white blood cell count, neutrophils, monocytes, hs-CRP, D-dimer, NLRP1, and fasting blood glucose (FBG) (Table 1, *P* < 0.05). Subsequent pairwise comparisons further confirmed significant differences in NLRP1 expression among the groups. The NLRP1 level was the highest in the AMI group, significantly higher than that in the HC group (Table 2, Figure 2, *Z* = −8.141, *P* < 0.0001) and the UA group (Table 2, Figure 2, *Z* = −2.944, *P* = 0.01). The NLRP1 level in the UA group was also significantly higher than that in the HC group (Table 2, Figure 2, *Z* = −4.100, *P* < 0.001).

**Table 1:** Comparison of baseline data among the three groups of populations.

Variables	HC (*n* = 60)	UA (*n* = 60)	AMI (*n* = 245)	*P*
Male, *n* (%)	37 (61.67)	43 (71.67)	185 (75.51)	0.097
Age (year)	63.00 (53.00–70.00)	66.00 (56.75–70.00)	64.00 (55.00–72.00)	0.472
Smoking, *n* (%)	18 (30.00)	24 (40.00)	85 (34.69)	0.515
Drinking, *n* (%)	14 (23.33)	14 (23.33)	46 (18.78)	0.596
Hypertension, *n* (%)	36 (60.00)	41 (68.33)	134 (54.69)	0.148
Diabetes, *n* (%)	11 (18.33)	21 (35.00)	74 (30.20)	0.104
BMI (kg/m^2^)	24.53 (22.62–27.43)	25.58 (23.48–27.68)	25.10 (23.08–27.06)	0.534
Leukocytes (×10^9^/L)	5.60 (4.57–6.73)	6.45 (5.57–7.90)	9.60 (7.70–11.70)	<0.001
Neutrophils (×10^9^/L)	3.35 (2.62–4.21)	4.03 (3.46–5.21)	7.82 (5.79–9.93)	<0.001
Lymphocytes (×10^9^/L)	1.60 (1.20–1.90)	1.65 (1.30–2.00)	1.20 (0.90–1.60)	<0.001
Monocytes (×10^9^/L)	0.36 (0.27–0.47)	0.40 (0.32–0.56)	0.50 (0.36–0.64)	<0.001
Platelets (×10^9^/L)	201 (166–223)	207 (178–241)	215 (178–261)	0.046
Hemoglobin (g/L)	142.08 ± 13.91	133.65 ± 16.50	133.65 ± 16.50	<0.001
hs-CRP (mg/L)	0.70 (0.50–1.54)	0.85 (0.50–3.23)	5.30 (1.40–15.40)	<0.001
TC (mmol/L)	4.33 (3.43–5.05)	4.04 (3.36–4.77)	4.22 (3.52–4.90)	0.561
TG (mmol/L)	1.45 (0.99–1.93)	1.66 (1.11–1.86)	1.38 (0.96–1.98)	0.374
HDL-C (mmol/L)	1.02 (0.89–1.28)	0.97 (0.86–1.27)	0.88 (0.76–1.02)	<0.001
LDL-C (mmol/L)	2.44 ± 0.94	2.38 ± 0.86	2.43 ± 0.79	0.901
FBG (mmol/L)	5.50 (5.10–6.29)	5.80 (5.16–6.81)	6.30 (5.42–8.30)	<0.001
TbIL (μmol/L)	11.45 (8.78–17.38)	12.10 (8.38–15.12)	11.70 (8.40–16.90)	0.956
Albumin (g/L)	43.47 ± 3.78	43.22 ± 3.44	38.40 ± 3.89	<0.001
BUN (mmol/L)	5.06 (4.34–5.99)	5.17 (4.45–6.01)	5.39 (4.33–6.54)	0.572
Scr (µmol/L)	61.50 (54.00–72.00)	66.50 (57.75–74.00)	62.00 (53.00–73.00)	0.354
UAc (μmol/L)	295 (244–345)	307 (256–365)	296 (238–361)	0.652
Fibrinogen (g/L)	2.57 (2.23–2.81)	2.96 (2.50–3.63)	2.70 (2.32–3.34)	0.005
D-dimer (mg/L)	0.10 (0.10–0.20)	0.15 (0.10–0.44)	0.49 (0.23–1.04)	<0.001
NLRP1 (pg/mL)	22.80 (13.09–35.93)	46.33 (32.99–73.53)	72.13 (35.33–201.38)	<0.001

HC group, healthy control group; UA group, patients with unstable angina pectoris Group; AMI group, patients with acute myocardial infarction group; BMI, body mass index; hs-CRP, high-sensitivity C-reactive protein; TC, total cholesterol; TG, triglyceride; HDL-C, high-density lipoprotein cholesterol; LDL-C, Low-density lipoprotein cholesterol; FBG, fasting blood glucose; TbIL, total bilirubin; BUN, blood urea nitrogen; Scr, serum creatinine; UAc, uric acid; NLRP1, NOD-like receptor protein 1; *P*, *P*-value.

**Table 2:** Comparative analysis of NLRP1 expression levels among the three groups.

Comparison group	Test value	SE	*Z*	*P*
HC-UA	−78.983	19.264	−4.100	<0.001
HC-AMI	−123.727	15.198	−8.141	<0.0001
UA-AMI	−44.744	15.198	−2.944	0.010

HC group, healthy control group; UA group, unstable angina group; AMI group, acute myocardial infarction group; SE, standard error; *Z*, *Z*-test; *P*, *P*-value.

### Comparison of clinical data between patients in the MACE and NMACE groups

The 245 AMI patients were divided into the MACE group (*n* = 59) and the NMACE group (*n* = 186) according to whether MACE events occurred during the half-year follow-up. No statistically significant differences were found between the two groups in general demographics and medical histories, such as gender, age, smoking, drinking, hypertension, and diabetes (Table 3, *P* > 0.05). Compared with the NMACE group, the MACE group had an increased leukocyte count, an increased neutrophil count, and a decreased lymphocyte count (Table 3, *P* < 0.05). Levels of hs-CRP, D-dimer, blood urea nitrogen (BUN), and serum creatinine (Scr) were significantly higher in the MACE group (*P* < 0.001). In addition, myocardial injury markers (hs-cTnT and NT-proBNP) were markedly increased in the MACE group, whereas left ventricular ejection fraction (LVEF) was significantly decreased (Table 3, *P* < 0.01).

**Table 3:** Comparison of baseline data between the NMACE group and the MACE group.

Variables	NMACE (*n* = 186)	MACE (*n* = 59)	Statistic	*P*
Male, *n* (%)	139 (74.73)	46 (77.97)	*χ*^2^ = 0.25	0.615
Age (year)	65.00 (56.00–72.00)	60.00 (54.00–73.50)	*Z* = −0.48	0.628
Smoking, *n* (%)	65 (34.95)	20 (33.90)	*χ*^2^ = 0.02	0.883
Drinking, *n* (%)	38 (20.43)	8 (13.56)	*χ*^2^ = 1.39	0.239
Hypertension, *n* (%)	103 (55.38)	31 (52.54)	*χ*^2^ = 0.15	0.703
Diabetes, *n* (%)	57 (30.65)	17 (28.81)	*χ*^2^ = 0.07	0.789
BMI (kg/m^2^)	25.39 (23.36–27.27)	24.22 (22.37–26.29)	*Z* = −1.90	0.057
Leukocytes (×10^9^/L)	9.40 (7.60–11.57)	10.00 (9.10–11.90)	*Z* = −2.11	0.035
Lymphocytes (×10^9^/L)	1.30 (1.00–1.70)	1.00 (0.80–1.20)	*Z* = −3.86	<0.001
Neutrophils (×10^9^/L)	7.36 (5.63–9.44)	8.48 (7.22–10.39)	*Z* = −2.87	0.004
Monocytes (×10^9^/L)	0.50 (0.36–0.64)	0.52 (0.38–0.70)	*Z* = −0.55	0.584
Platelets (×10^9^/L)	215.00 (181.25–258.50)	219.00 (168.50–267.00)	*Z* = −0.13	0.898
Hemoglobin (g/L)	134.92 ± 17.09	129.64 ± 13.85	*t* = 2.16	0.032
hs-CRP (mg/L)	3.45 (1.10–11.62)	13.70 (3.35–34.50)	*Z* = −4.91	<0.001
Fibrinogen (g/L)	2.65 (2.30–3.28)	2.92 (2.37–3.54)	*Z* = −1.46	0.144
D-dimer (mg/L)	0.40 (0.21–0.85)	0.78 (0.38–1.47)	*Z* = −3.39	<0.001
TC (mmol/L)	4.33 (3.58–4.92)	3.97 (3.17–4.83)	*Z* = −1.54	0.125
TG (mmol/L)	1.42 (1.00–1.98)	1.30 (0.94–1.83)	*Z* = −0.83	0.409
HDL-C (mmol/L)	0.89 (0.76–1.02)	0.87 (0.77–1.01)	*Z* = −0.16	0.876
LDL-C (mmol/L)	2.50 (2.02–3.01)	2.26 (1.73–2.80)	*Z* = −1.88	0.06
ApoA1 (g/L)	1.15 (1.01–1.29)	1.17 (0.98–1.28)	*Z* = −0.74	0.461
ApoB (g/L)	0.90 ± 0.25	0.84 ± 0.26	*t* = 1.65	0.101
Lipoprotein (a) (mg/L)	207.00 (130.75–388.75)	250.00 (101.00–357.50)	*Z* = −0.08	0.939
FBG (mmol/L)	6.04 (5.37–8.64)	6.71 (5.64–8.09)	*Z* = −1.29	0.197
HbAlC (%)	6.40 (5.50–7.57)	6.36 (5.68–7.96)	*Z* = −0.64	0.521
Albumin (g/L)	38.40 (36.30–41.00)	37.50 (35.85–39.60)	*Z* = −1.46	0.143
TbIL (μmol/L)	11.60 (8.40–16.70)	12.70 (8.40–18.40)	*Z* = −0.75	0.454
BUN (mmol/L)	5.09 (4.15–6.20)	5.94 (5.00–7.55)	*Z* = −3.77	<0.001
Scr (µmol/L)	60.00 (52.00–70.00)	69.00 (58.50–88.50)	*Z* = −3.35	<0.001
UAc (μmol/L)	286.50 (239.25–354.25)	323.00 (237.00–398.50)	*Z* = −1.72	0.085
Peak CK-MB (ng/mL)	63.90 (17.23–192.75)	105.00 (24.30–297.50)	*Z* = −1.92	0.055
Peak hs-cTnT (pg/mL)	2,119.50 (917.25–4,389.75)	5,163.00 (3,062.00–9,287.00)	*Z* = −4.68	<0.001
Peak NT-proBNP (pg/mL)	1,166.00 (532.75–2,382.96)	3,452.00 (1,610.50–6,383.00)	*Z* = −5.88	<0.001
LVEF (%)	54.80 (50.00–59.75)	51.00 (46.18–54.12)	*Z* = −2.89	0.004
STEMI, *n* (%)	119 (63.98)	39 (66.10)	*χ*^2^ = 0.09	0.767
Killip class II–IV, *n* (%)	20 (10.75)	6 (10.17)	*χ*^2^ = 0.02	0.899
Antilipidemic, *n* (%)	25 (13.44)	7 (11.86)	*χ*^2^ = 0.10	0.754
Antiplatelet, *n* (%)	31 (16.67)	7 (11.86)	*χ*^2^ = 0.79	0.375
Antihypertensive, *n* (%)	45 (24.19)	14 (23.73)	*χ*^2^ = 0.01	0.942
SII	1,224.82 (756.15–1,959.21)	1,759.58 (1,257.44–2,643.32)	*Z* = −3.77	<0.001
SIRI	2.62 (1.59–4.74)	4.04 (2.84–6.67)	*Z* = −3.92	<0.001
NLRP1 (pg/mL)	58.81 (32.23–169.58)	150.91 (81.88–435.06)	*Z* = −5.04	<0.001

NMACE, non-major adverse cardiovascular events; MACE, major adverse cardiovascular events; BMI, body mass index; hs-CRP; high-sensitivity C-reactive protein; TC, total cholesterol; TG, triglyceride; HDL-C, high-density lipoprotein cholesterol; LDL-C, low-density lipoprotein cholesterol; FBG, fasting blood glucose; HbAlC, glycated hemoglobin; TbIL, total bilirubin; BUN, blood urea nitrogen; LVEF, left ventricular ejection fraction; Scr, serum creatinine; UAc, uric acid; peak CK-MB, peak creatine kinase-MB isoenzyme; peak hs-cTnT, peak high-sensitivity cardiac troponin T; peak NT-proBNP, peak N-terminal pro-brain natriuretic peptide; STEMI, ST-segment elevation myocardial infarction; SII, systemic inflammatory index; SIRI, systemic inflammatory response index; NLRP1, NOD-like receptor protein 1; *P*, *P*-value.

**Figure 3:**
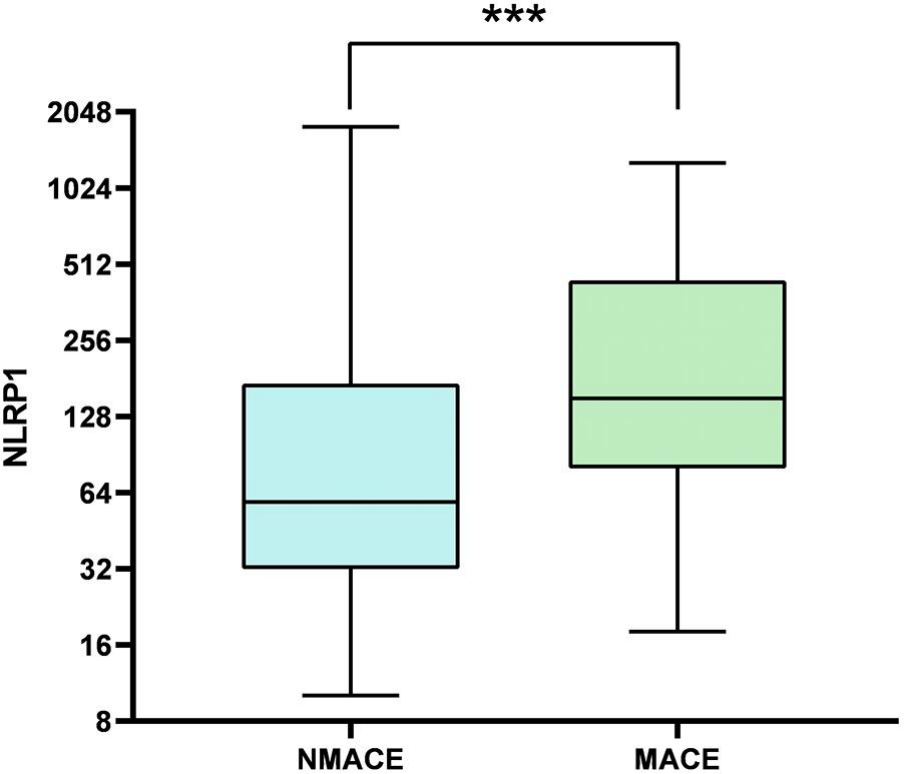
Comparison of box plots of NLRP1 levels between the MACE group and the NMACE group. There was a significant difference in NLRP1 levels between the two groups (*P* < 0.001); NMACE, non-major adverse cardiovascular events; MACE, major adverse cardiovascular events; NLRP1, NOD-like receptor protein 1.

Furthermore, the levels of systemic inflammatory indicators, namely, SII, SIRI, and NLRP1, were significantly higher in the MACE group (Table 3, Figure 3, *P* < 0.05).

### Correlation between NLRP1 and other indicators

**Figure 4:**
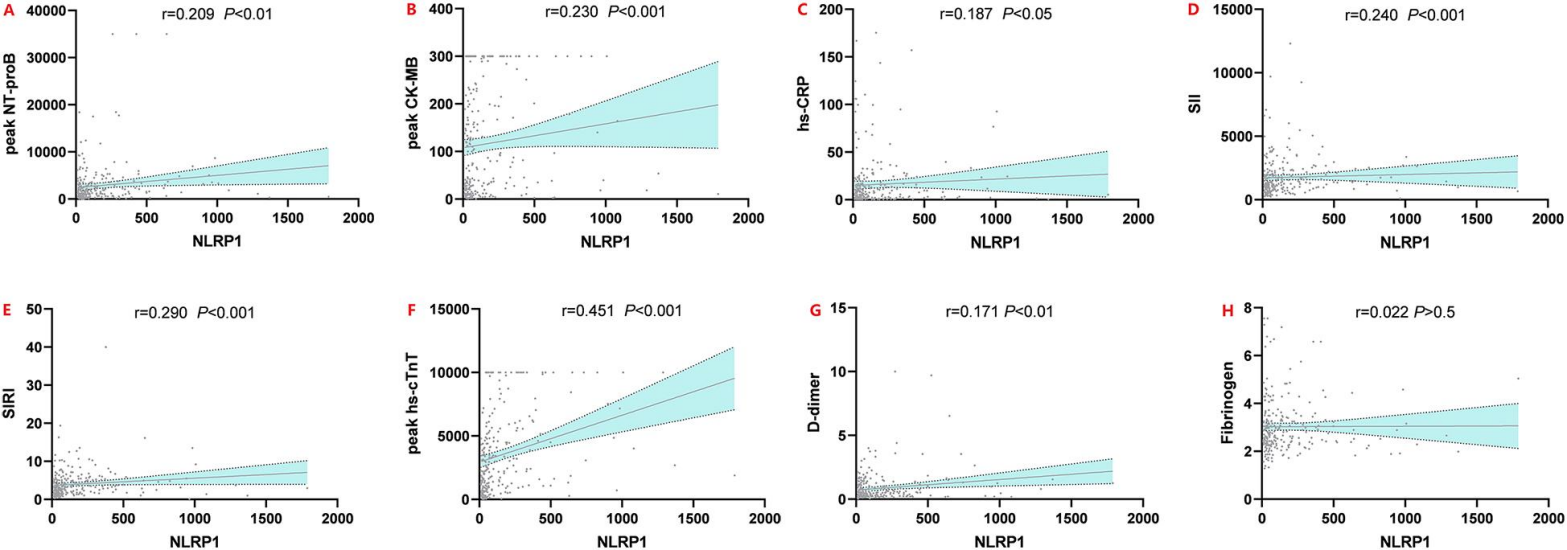
Spearman rank correlation scatter plots of NLRP1 and six indicators. Peak NT-proBNP, peak N-terminal pro-brain natriuretic peptide; peak CK-MB, peak creatine kinase-MB isoenzyme; hs-CRP, high-sensitivity C-reactive protein; peak hs-cTnT, peak high-sensitivity cardiac troponin T; SII, systemic inflammatory index; SIRI, systemic inflammatory response index; NLRP1, NOD-like receptor protein 1.

Spearman correlation analysis revealed that serum NLRP1 levels in AMI patients were significantly positively correlated with multiple clinical indicators (Figure 4, *P* < 0.05). Specifically, weak positive correlations were observed with hs-CRP (*r* = 0.158, *P* < 0.05), peak hs-cTnT (*r* = 0.161, *P* < 0.05), and D-dimer (*r* = 0.171, *P* < 0.01); moderate positive correlations with peak CK-MB (Figure 4, *r* = 0.265, *P* < 0.001), SII (*r* = 0.234, *P* < 0.001), and peak NT-proBNP (*r* = 0.255, *P* < 0.001); and the strongest positive correlation with SIRI (*r* = 0.389, *P* < 0.001). No significant correlation was found between NLRP1 and fibrinogen (*r* = 0.022, *P* > 0.05). These results suggest that NLRP1 expression can reflect the intensity of the inflammatory response and the extent of myocardial injury in AMI patients, indicating its potential clinical value.

### LASSO-based multivariable logistic regression analysis

**Figure 5:**
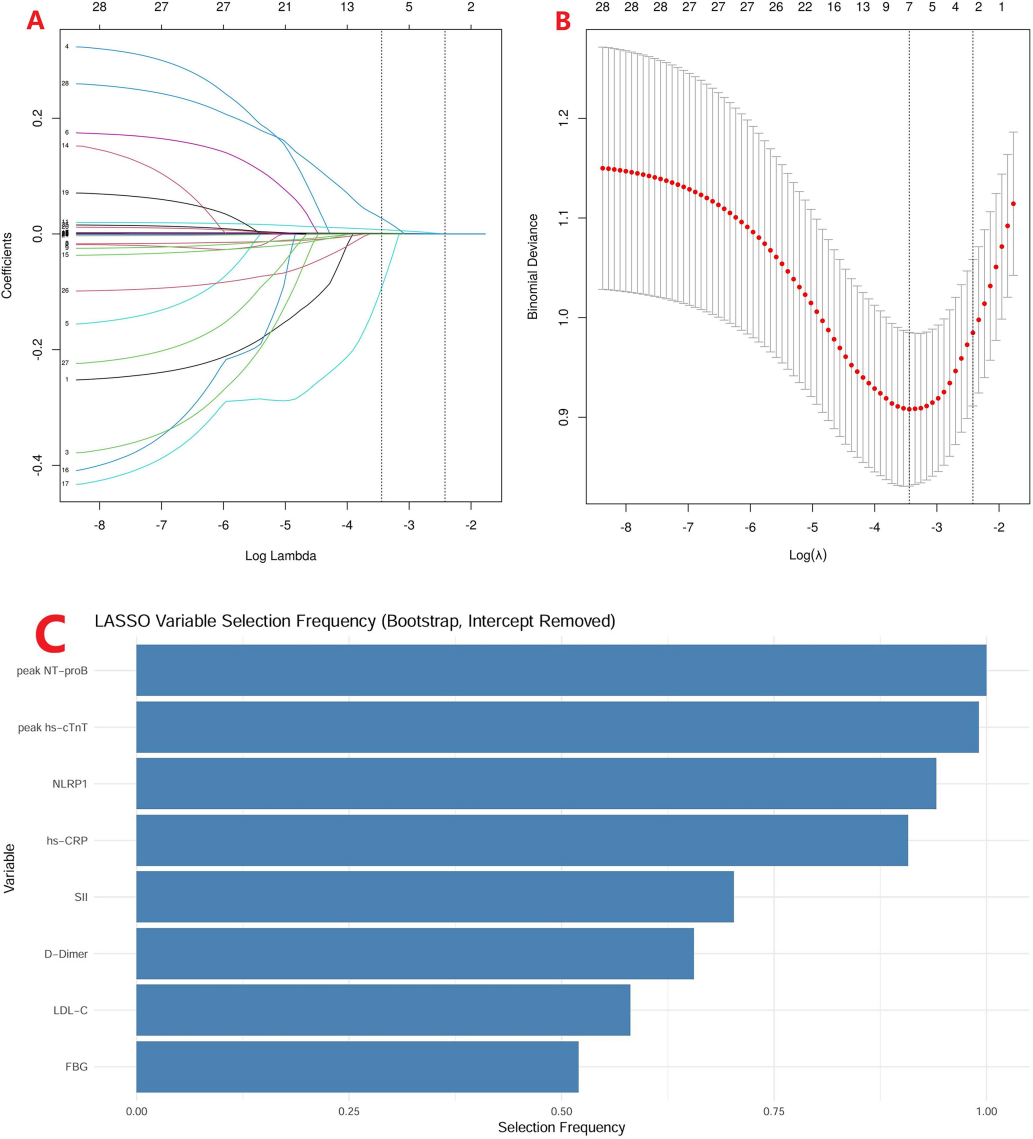
LASSO regression with bootstrap validation. (**A**) LASSO coefficient profiles of candidate variables across a sequence of log(*λ*) values. (**B**) Ten-fold cross-validation curve showing the binomial deviance for different *λ* values, with the optimal *λ* indicated by the dotted line. (**C**) Selection frequency of variables in 1,000 bootstrap replications, highlighting eight variables (peak NT-proBNP, peak hs-cTnT, NLRP1, hs-CRP, SII, D-dimer, LDL-C, and FBG) consistently identified as candidate predictors.

To address the risk of model overfitting, we first performed LASSO regression with 1,000 bootstrap replications, which identified eight candidate predictors (Figure 5; FBG, LDL-C, D-dimer, SII, hs-CRP, NLRP1, peak hs-cTnT, and peak NT-proBNP). These variables were subsequently entered into a multivariable logistic regression model for 6-month MACE. With 59 events, the events per variable (EPV) for the full model was approximately 7.4, slightly below the conventional threshold of 10. Notably, only four variables—hs-CRP, NLRP1, peak hs-cTnT, and peak NT-proBNP—remained independent predictors in the multivariable analysis. In addition, no evidence of multicollinearity was detected among these four predictors (all variance inflation factors (VIFs) <2). Because the OR of NLRP1 per unit was close to 1.0, we further expressed its effect size using standardized increments: per SD (250.7 pg/mL) and per IQR (166.1 pg/mL). This transformation yielded more interpretable results, with ORs of 1.49 (95% CI: 1.09–2.05) per SD and 1.30 (95% CI: 1.06–1.61) per IQR, respectively (Table 4).

**Table 4:** Multivariable logistic regression analysis.

Variables	*β*	SE	*Z*	OR (95% CI)	*P*
FBG (mmol/L)	−0.09	0.06	−1.48	0.92 (0.81–1.03)	0.139
LDL-C (mmol/L)	−0.43	0.25	−1.73	0.65 (0.40–1.06)	0.084
D-dimer (mg/L)	0.13	0.15	0.84	1.14 (0.85–1.52)	0.398
SII	0.00	0.00	1.28	1.00 (1.00–1.00)	0.200
hs-CRP	0.01	0.01	2.58	1.02 (1.01–1.03)	0.010
Peak hs-cTnT (pg/mL)	0.01	0.00	3.35	1.01 (1.01–1.01)	<0.001
Peak NT-proBNP (pg/mL)	0.01	0.00	3.47	1.01 (1.01–1.01)	<0.001
NLRP1 (pg/mL)^a^	0.01	0.00	2.48	1.01 (1.01–1.01)	0.013
NLRP1 per SD^a^	0.40	0.16	2.48	1.49 (1.09–2.05)	0.013
NLRP1 per IQR^a^	0.27	0.11	2.48	1.30 (1.06–1.61)	0.013

FBG, fasting blood glucose; LDL-C, low-density lipoprotein cholesterol; hs-CRP, high-sensitivity C-reactive protein; peak hs-cTnT, peak high-sensitivity cardiac troponin T; peak NT-proBNP, peak N-terminal pro-brain natriuretic peptide; NLRP1, NOD-like receptor protein 1; SD, standard deviation (≈251 pg/mL); IQR, interquartile range (≈166 pg/mL). *β,* regression coefficient; SE, standard error of *β*; *Z*, Wald *Z* statistic (*β/*SE); OR, odds ratio; *P*, *P*-value.

^a^ NLRP1 and its different expression forms (per SD, per IQR) are all different scale treatments of the same index, which are respectively analyzed by a regression model, but not simultaneously included in the same multifactor model.

### Analysis of ROC and RCS curves

**Figure 6:**
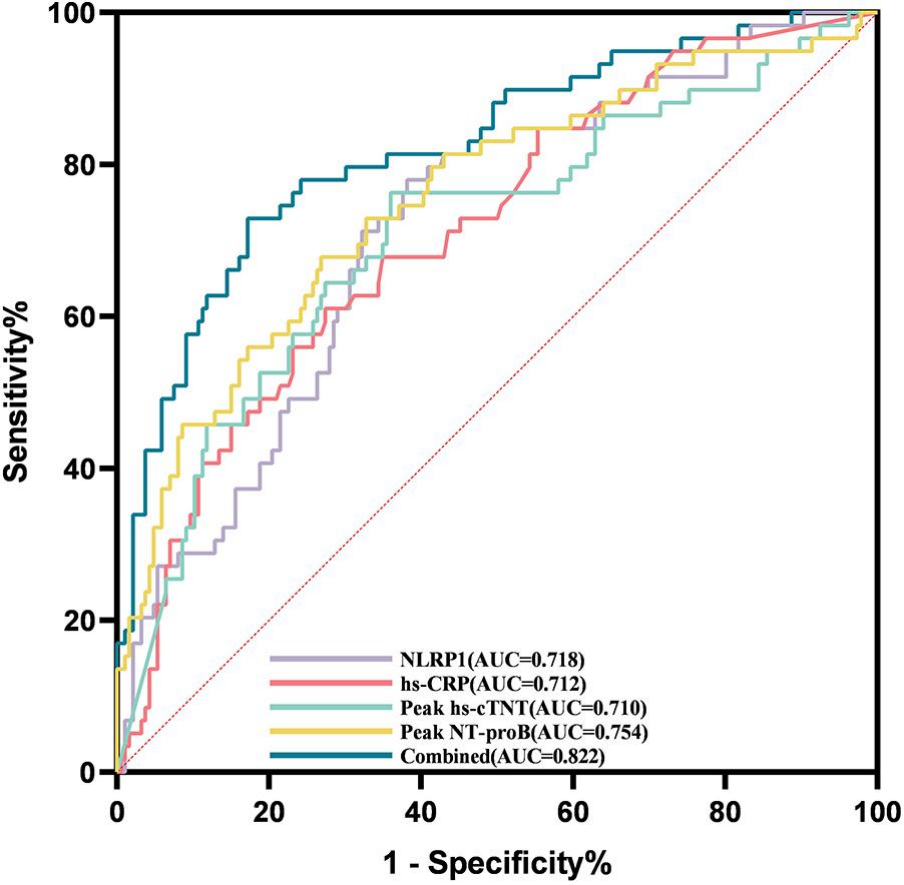
ROC curves for evaluating the MACE events within half a year in patients with AMI by NLRP1, hs-CRP, peak NT-proBNP, peak hs-cTnT alone, and their combined detection. Combined, area under the ROC curve of the combination of NLRP1, peak NT-proBNP, peak hs-cTnT, and hs-CRP. Peak NT-proBNP, peak N-terminal pro-brain natriuretic peptide; hs-CRP, high-sensitivity C-reactive protein; peak hs-cTnT, peak high-sensitivity cardiac troponin T; NLRP1, NOD-like receptor protein 1; AUC, area under the curve.

ROC curve analysis showed that the AUC of NLRP1 alone for predicting MACE was 0.718 (Figure 6, Table 5, 95% CI: 0.645–0.791, *P* < 0.001), with a cutoff value of 76.87 pg/mL, a sensitivity of 0.78, and a specificity of 0.62. The DeLong test indicated that there was no significant difference in its AUC compared with the individual detections of hs-CRP, peak NT-proBNP, and peak hs-cTnT (Table 6, *P* > 0.05). However, the AUC of the combined detection of NLRP1 with the above three markers reached 0.822. Meanwhile, the AUC of the combined index was also significantly higher than that of the individual detections of hs-CRP (Table 6, ΔAUC = −0.110, *P* = 0.008), peak hs-cTnT (Table 6, ΔAUC = −0.112, *P* < 0.001), and peak NT-proBNP (Table 6, ΔAUC = −0.068, *P* = 0.019).

**Table 5:** ROC curve analysis.

Variables	AUC	95% CI	*P*	Cutoff	Sensitivity	Specificity
NLRP1	0.718	(0.645–0.791)	<0.001	76.87	0.78	0.62
Peak hs-cTnT	0.710	(0.630–0.790)	<0.001	3,038.50	0.76	0.64
Peak NT-proBNP	0.754	(0.680–0.829)	<0.001	2,202.00	0.68	0.73
Peak hs-CRP	0.712	(0.637–0.787)	<0.001	10.05	0.61	0.73

AUC, area under the curve; 95% CI, 95% confidence interval; *P*, *P*-value; cutoff, cutoff value; peak CK-MB, peak creatine kinase-MB isoenzyme; peak hs-cTnT, peak high-sensitivity cardiac troponin T; peak NT-proBNP, peak N-terminal pro-brain natriuretic peptide.

**Table 6:** Pairwise sample area differences under the ROC curve.

Inspection results	ΔAUC	Standard error value	95% CI	*P*
NLRP1—hs-CRP	0.006	0.274	−0.093 to 0.106	0.903
NLRP1—peak NT-proBNP	−0.036	0.275	−0.138 to 0.065	0.486
NLRP1—peak hs-cTnT	0.008	0.278	−0.078 to 0.094	0.857
Peak NT-proBNP—peak hs-cTnT	0.044	0.280	−0.05 to 0.138	0.358
Peak NT-proBNP—combined	−0.068	0.264	−0.125 to −0.011	0.019
Peak hsTnT—combined	−0.112	0.269	−0.171 to −0.053	<0.001
NLRP1—combined	−0.104	0.264	−0.185 to −0.024	0.011
hs-CRP—combined	−0.110	0.266	−0.192 to −0.029	0.008

Combined, area under the ROC curve of the combination of NLRP1, peak NT-proBNP, peak hs-cTnT, and hs-CRP; hs-CRP, high-sensitivity C-reactive protein; peak CK-MB, peak creatine kinase-MB isoenzyme; peak hs-cTnT, peak high-sensitivity cardiac troponin T; peak NT-proBNP, peak N-terminal pro-brain natriuretic peptide; NLRP1, NOD-like receptor protein 1; 95% CI, 95% confidence interval; AUC, area under the curve; ΔAUC, AUC difference; *P*, *P*-value.

To further explore whether the prognostic value of NLRP1 differed across patient subgroups, we performed stratified analyses based on biomarker cutoff values. As shown in Table 7, adding NLRP1 to the baseline model improved AUC across most subgroups. The improvements were statistically significant in low peak hs-cTnT (<3,038.5) (ΔAUC = 0.055, *P* = 0.047), low hs-CRP (<10.05) (ΔAUC = 0.038, *P* = 0.048), and high SII (≥1,200.88) (ΔAUC = 0.049, *P* = 0.026). Other subgroups showed modest upward trends, but not statistically significant. These results suggest that the incremental prognostic value of NLRP1 is particularly evident in patients with lower levels of myocardial injury (low peak hs-cTnT), lower systemic inflammation (low peak hs-CRP), or high immune-inflammatory burden as captured by SII.

**Table 7:** Stratified analyses by cutoff values.

Stratification	AUC (base)	AUC (base + NLRP1)	ΔAUC	95% CI	*P*
High peak hs-cTnT (≥3,038.5)	0.827	0.847	0.020	−0.020 to 0.065	0.166
Low peak hs-cTnT (<3,038.5)	0.732	0.786	0.055	−0.012 to 0.139	0.047
High peak NT-proBNP (≥2,202.0)	0.822	0.829	0.007	−0.008 to 0.025	0.191
Low peak NT-proBNP (<2,202.0)	0.670	0.728	0.058	−0.008 to 0.136	0.051
High hs-CRP (≥10.05)	0.772	0.788	0.016	−0.018 to 0.053	0.170
Low hs-CRP (<10.05)	0.765	0.803	0.038	−0.003 to 0.085	0.048
High SIRI (≥3.06)	0.846	0.853	0.007	−0.013 to 0.030	0.266
Low SIRI (<3.06)	0.672	0.732	0.060	−0.022 to 0.160	0.089
High SII (≥1,200.88)	0.769	0.818	0.049	−0.000 to 0.106	0.026
Low SII (<1,200.88)	0.811	0.804	−0.007	−0.031 to 0.014	0.269

Peak hs-cTnT, peak high-sensitivity cardiac troponin T; peak NT-proBNP, peak N-terminal pro-brain natriuretic peptide; hs-CRP, high-sensitivity C-reactive protein; NLRP1, NOD-like receptor protein 1; AUC, area under the curve; ΔAUC, AUC difference; 95% CI, 95% confidence interval; *P*, *P*-value.

**Figure 7:**
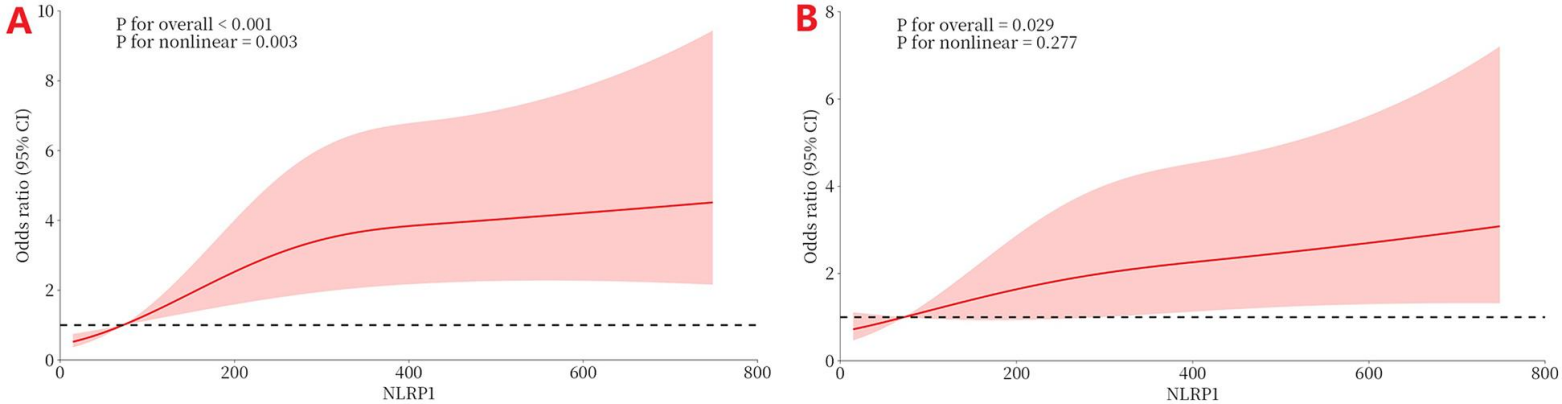
The dose–response relationship between NLRP1 and the risk of MACE (RCS). (**A**) Without covariate adjustment (directly fitting the association between NLRP1 and MACE). (**B**) Adjusting for significant covariates (hs-CRP, peak hs-cTnT, peak NT-proBNP) selected in the multivariable regression. NLRP1, NOD-like receptor protein 1; 95% CI, 95% confidence interval.

Figure 7 shows the RCS curve, analyzing the dose–response relationship between NLRP1 levels and the risk of MACE. In the model without covariate adjustment (Figure 7A), there was a significant overall association between NLRP1 levels and adverse prognostic events (Figure 7A, *P* < 0.001), and this association had significant non-linear characteristics (*P* = 0.003 for the non-linear test). After further adjusting for hs-CRP, hs-cTnT, and NT-proBNP in the model (Figure 7B), the overall association between NLRP1 and prognosis remained statistically significant (*P* = 0.029), but the non-linear characteristics were no longer significant (*P* = 0.277 for the non-linear test).

### Incremental predictive value and clinical benefit of NLRP1

**Figure 8:**
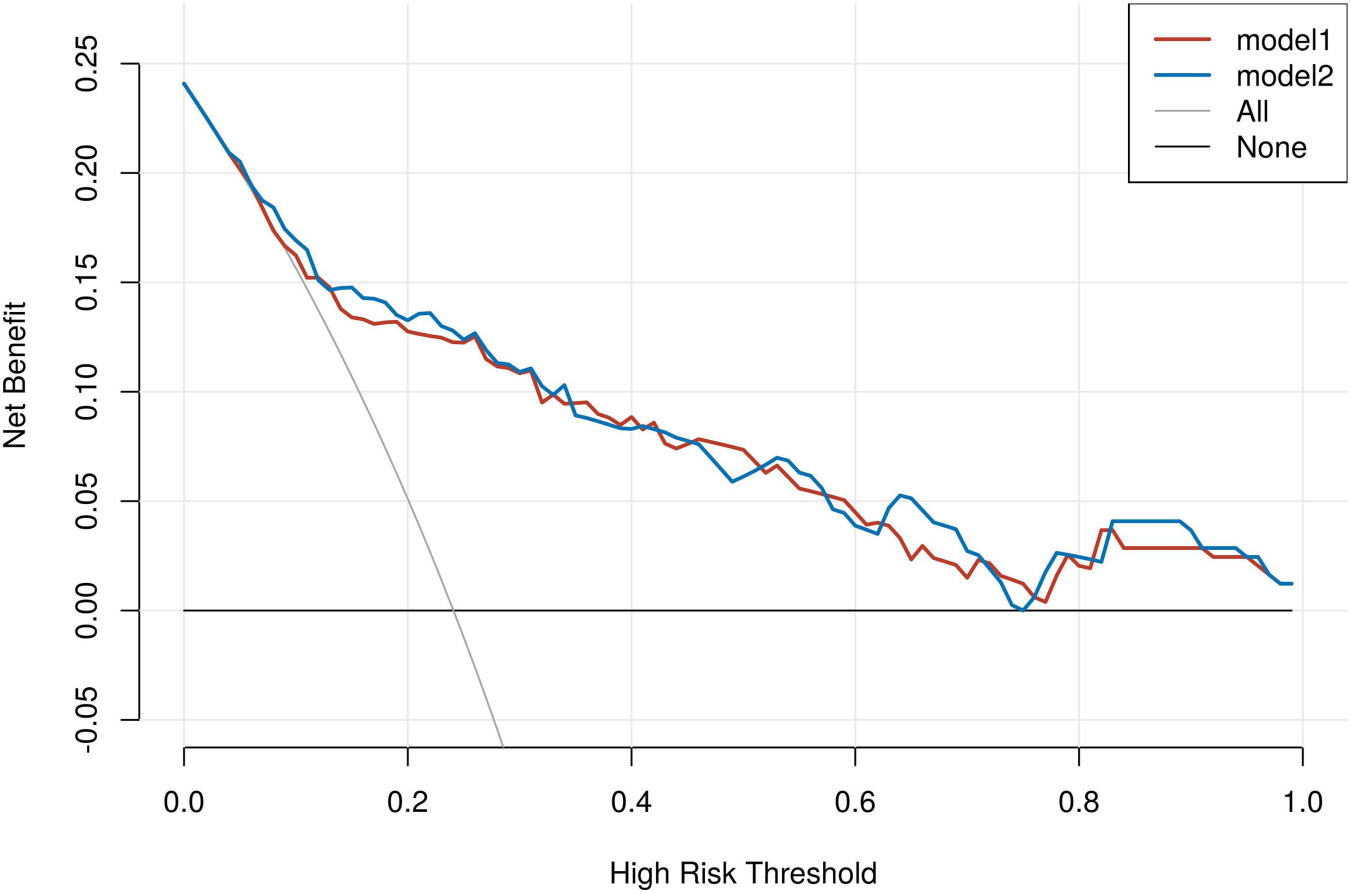
Decision curve analysis comparing baseline and NLRP1-enhanced models for MACE in AMI. None, treat-none strategy, assuming that no patients receive intervention; ALL, treat-all strategy, assuming that all patients receive intervention regardless of risk; Model 1, baseline model including hs-CRP, hs-cTnT, and NT-proBNP; Model 2, Model 1 plus NLRP1.

To further evaluate the incremental predictive value of NLRP1 beyond established biomarkers, we calculated the IDI and the category-free NRI. As shown in Table 8, the IDI was close to zero (IDI = 0.000, 95% CI: 0.003–0.004, *P* = 0.834), suggesting no significant overall improvement in mean discrimination. In contrast, the NRI demonstrated a positive net reclassification (NRI = 0.315, 95% CI: 0.020–0.617, *P* = 0.037), indicating that the addition of NLRP1 reclassified a considerable proportion of patients into more appropriate risk categories. These findings provide complementary information to the ROC and RCS analyses presented above. In addition, DCA showed that both models yielded comparable net benefits across most threshold probabilities. Importantly, the model incorporating NLRP1 provided a modestly higher net benefit than the baseline model within the clinically relevant threshold range of approximately 0.10–0.35, suggesting potential incremental clinical utility of NLRP1 for risk stratification (Figure 8).

**Table 8:** IDI and NRI for evaluating the added value of NLRP1.

Metric	Point estimate	95% CI	*P*
IDI	0.000	0.003–0.004	0.834
NRI	0.315	0.020–0.617	0.037

IDI, integrated discrimination improvement; NRI, net reclassification improvement; 95% CI, 95% confidence interval; *P*, *P*-value.

### NLRP1 grouping based on ROC cutoff values

In this study, the data were divided into two groups based on the cutoff value of the Youden index: the low-NLRP1 group (*n* = 128) and the high-NLRP1 group (*n* = 117). Multiple indicators of the two groups were compared and analyzed. There were no statistically significant differences between the two groups in terms of age, gender, diabetes, smoking, and the proportion of ST-elevation myocardial infarction (STEMI) (Table 9, *P* > 0.05), indicating that the two groups of people were relatively balanced in these basic characteristics, which could exclude the interference of these factors on the subsequent results to some extent. In terms of indicators related to inflammation and myocardial injury, such as hs-CRP, peak hs-cTnT, and peak NT-proBNP, SII, and SIRI, the values in the high-NLRP1 group were all higher than those in the low-NLRP1 group, and the differences were statistically significant (Table 9, *P* < 0.05).

**Table 9:** Differences in characteristics and indicators between the high- and low-level groups of NLRP1 under the Youden index grouping.

Variables	Low-level group (*n* = 128)	High-level group (*n* = 117)	Statistic	*P*
Age (year)	62.00 (54.00–72.00)	66.00 (56.00–73.00)	*Z* = −1.23	0.218
Male, *n* (%)	100 (78.12)	85 (72.65)	*χ*^2^ = 0.99	0.319
Smoking, *n* (%)	48 (37.50)	37 (31.62)	*χ*^2^ = 0.93	0.334
Diabetes, *n* (%)	42 (32.81)	32 (27.35)	*χ*^2^ = 0.87	0.352
STEMI, *n* (%)	83 (64.84)	75 (64.10)	*χ*^2^ = 0.01	0.904
hs-CRP (mg/L)	3.45 (1.08–10.88)	8.20 (2.10–23.60)	*Z* = −3.09	0.002
Peak hs-cTnT (pg/mL)	1,482.00 (594.25–3,857.25)	4,016.00 (1,899.00–7,928.00)	*Z* = −6.11	<0.001
Peak NT-proBNP (pg/mL)	1,175.50 (553.25–2,736.75)	1,811.00 (924.00–3,459.00)	*Z* = −2.24	0.025
SII	1,210.71 (682.39–1,872.17)	1,547.62 (974.70–2,448.13)	*Z* = −3.17	0.002
SIRI	2.43 (1.42–4.35)	3.76 (2.37–5.49)	*Z* = −3.98	<0.001
MACE, *n* (%)	23 (17.97)	36 (30.77)	*χ*^2^ = 5.48	0.019

hs-CRP, high-sensitivity C-reactive protein; peak hs-cTnT, peak high-sensitivity cardiac troponin T; peak NT-proBNP, peak N-terminal pro-brain natriuretic peptide; SII, systemic inflammatory index; SIRI, systemic inflammatory response index; MACE, non-major adverse cardiovascular events; *P*, *P*-value.

## Discussion

The inflammatory response plays a pivotal role in the pathological process and prognosis of AMI. As a core regulatory molecule of the inflammasome, the clinical significance of NLRP1 in AMI has not been systematically elucidated. This study systematically explored the association between NLRP1 and AMI for the first time and found that the serum NLRP1 level gradually increased with the severity of coronary heart disease, from the healthy controls to UA and then to AMI. Serum NLRP1 was closely related to the inflammatory activity and the degree of myocardial injury in AMI patients. Importantly, NLRP1 is an independent risk factor for short-term MACE in AMI patients. Its efficacy in predicting MACE alone is reliable, and the combination with traditional inflammatory and myocardial injury markers can further improve the accuracy of risk stratification. These findings reveal the potential role of NLRP1 in the inflammatory response and prognosis assessment of AMI, providing new biological clues for optimizing the risk assessment of AMI patients. The results indicate that NLRP1 can not only reflect the inflammatory level of AMI patients but also has the potential to serve as a biomarker for predicting poor prognosis, suggesting important clinical application prospects.

The elevated expression of NLRP1 in patients with AMI and its association with poor prognosis can be attributed to the inflammatory regulatory mechanism in which it participates. This study demonstrated that the serum NLRP1 level in AMI patients was significantly higher compared with those with UA and healthy individuals. Moreover, NLRP1 levels were closely correlated with inflammatory markers such as hs-CRP and the SIRI, as well as myocardial injury indicators including hs-cTnT and NT-proBNP. This is consistent with the biological function of NLRP1 as the core component of the inflammasome. By activating caspase-1, NLRP1 mediates the maturation and release of pro-inflammatory cytokines, such as IL-1β and IL-18, thereby driving local myocardial inflammatory response and systemic immune activation (7, 9, 29). The present study expands on previous findings demonstrating an association between NLRP1 and the severity of coronary artery lesions in patients with unstable angina (15). Our results indicate that NLRP1 levels increase with the progression of coronary heart disease, from healthy individuals to those with unstable angina to those with acute myocardial infarction. This suggests that NLRP1 may contribute to disease progression by persistently amplifying inflammatory signaling, which aligns with the well-established principle that the magnitude of the inflammatory response influences the prognosis of acute myocardial infarction (16, 17). The strong correlation between NLRP1 and SIRI (*r* = 0.389) in this study may be related to the regulation of immune cell function by NLRP1. As a key molecule of the inflammasome, NLRP1 can activate downstream inflammatory pathways by sensing damage-associated molecular patterns (DAMPs) released from myocardial ischemia and necrosis, promote neutrophil infiltration and monocyte activation, and thus exacerbate systemic inflammatory imbalance (18). In addition, multivariate regression analysis shows that NLRP1 is an independent risk factor for MACE (OR = 1.01), and its predictive value is further improved after combining with traditional markers (AUC = 0.822). This may be because NLRP1 integrates information on both local myocardial injury (related to hs-cTnT and NT-proBNP) and systemic inflammation (related to hs-CRP and SIRI), reflecting the pathological state of AMI patients more comprehensively than a single marker.

From a broader clinical and mechanistic perspective, the prognostic value of NLRP1 is not limited to AMI. Gonzalez-Hidalgo et al. (19) found that the activation of NLRP1 has strict ligand specificity and tissue restriction, and its mechanism of action in cardiovascular tissues differs significantly from that of NLRP3. For example, NLRP1 is selectively upregulated in aortic occlusive lesions, while NLRP3 is predominant in aneurysmal lesions. This specificity positions it as a potential target molecule for fibroproliferative vascular diseases. The redox-sensitive regulatory mechanism of NLRP1, including the inhibitory effect mediated by thioredoxin (TRX), underpins its role in the cross-regulation of metabolic stress and inflammation in ischemic myocardium (20, 21). Furthermore, the proteolytic regulation of its function to find domain (FIIND) domain by dipeptidyl peptidase 9 (DPP9) presents a promising target for intervention strategies (22, 23).

In the prognostic assessment of AMI, traditional biomarkers such as NT-proBNP, hs-cTnT, and hs-CRP have been widely proven to be of great value. The results of this study further support their core position in the risk stratification of AMI patients. As a sensitive indicator of ventricular wall pressure load, an elevated level of NT-proBNP reflects the degree of ventricular remodeling and cardiac function impairment after myocardial infarction (24). The peak concentration of NT-proBNP was significantly elevated (*P* < 0.001) in the MACE group and moderately correlated with NLRP1 (*r* = 0.255), suggesting NLRP1 may contribute to ventricular remodeling by modulating the inflammatory response. As a specific biomarker of myocardial cell necrosis, hs-cTnT can be used to diagnose AMI and assess the degree of myocardial injury (25). This study found a weak correlation between hs-cTnT and NLRP1 (*r* = 0.161), and both were independent risk factors for MACE (OR = 1.01, *P* < 0.05), indicating that myocardial necrosis and inflammatory activation have a synergistic effect on the prognosis of AMI, which is consistent with the mechanism of inflammation exacerbating myocardial cell death in previous studies (6). As a classic systemic inflammatory biomarker, the level of hs-CRP is closely related to the instability of atherosclerotic plaques and the risk of cardiovascular events (26–28). In this study, the weak correlation between hs-CRP and NLRP1 (*r* = 0.158) and the improvement of the combined predictive efficacy (AUC = 0.822) suggest that NLRP1 can complement the deficiency of hs-CRP in the assessment of local inflammatory response. In addition, a weak but significant correlation was observed between NLRP1 and D-dimer (*r* = 0.171, *P* < 0.01), whereas no correlation was found with fibrinogen. This indicates that NLRP1 may be selectively linked to coagulation and fibrinolytic activity, rather than reflecting all coagulation parameters, further highlighting its multifaceted role in AMI prognosis. Notably, the RCS analysis initially suggested a non-linear relationship between NLRP1 and MACE risk, but this disappeared after adjusting for key covariates (hs-cTnT, NT-proBNP, hs-CRP). This indicates that the apparent non-linearity was largely due to confounding by these correlated biomarkers, and the true relationship between NLRP1 and MACE is approximately linear.

This study concludes that NLRP1 plays a role in the pathology of AMI by modulating the inflammatory response, with its serum levels potentially indicating disease severity and prognosis. Given its distinct molecular regulatory mechanisms, such as DPP9-dependent self-inhibition and redox switch, along with its tissue-specific effects, NLRP1 offers a novel perspective on the intricate network of cardiovascular inflammation. Furthermore, it provides preclinical evidence for developing precision treatments targeting inflammasomes, including DPP9 modulators, TRX mimetics, or IL-1β. From a clinical and economic perspective, however, it is important to consider the cost-effectiveness of introducing NLRP1 into routine practice. Conventional biomarkers such as hs-cTnT and NT-proBNP are widely available and relatively inexpensive, whereas the measurement of NLRP1 currently requires specialized assays and additional resources. Thus, the clinical application of NLRP1 should not be viewed as a substitute but as a complement, particularly in patient subgroups where traditional biomarkers show limitations (e.g., low hs-cTnT or low SIRI). In these settings, the incremental prognostic value of NLRP1 may justify the additional testing costs by enabling more precise risk stratification and potentially guiding personalized therapy, ultimately improving patient outcomes and optimizing healthcare resource allocation.

## Limitations

This study has several limitations. First, as a single-center investigation, all participants were from the Affiliated Hospital of Xuzhou Medical University, with a limited sample size of 245 AMI patients. This may introduce regional or population selection bias, necessitating validation through multicenter, large-sample studies. Second, the follow-up period was only six months, preventing assessment of NLRP1's predictive value for long-term AMI prognosis, such as MACE risk beyond one year. Third, only serum NLRP1 levels were measured, without exploring its expression and localization in myocardial tissues. In addition, the mechanism analysis relied primarily on existing literature, lacking *in vitro* and *in vivo* experimental validation. Finally, this study did not directly assess thrombus burden, but instead used D-dimer and fibrinogen as surrogate indicators, which may not fully reflect the complexity of thrombotic status in AMI patients.

## Conclusion

Serum NLRP1 levels increase with the severity of coronary artery disease (from healthy controls to UA to AMI). In AMI patients, it is closely associated with inflammatory response and myocardial injury, and is an independent risk factor for short-term MACE. Combining it with traditional markers can improve the efficacy of prognostic assessment.

## Data Availability

The raw data supporting the conclusions of this article will be made available by the authors, without undue reservation.
